# Diagnostic Stewardship to Optimize Blood Culture in a Pediatric Cardiac Intensive Care Unit

**DOI:** 10.1097/pq9.0000000000000887

**Published:** 2026-06-05

**Authors:** Jun Sasaki, Angela Sorensen, Kristen Ferlisi, Lauren Shaw, Maryellen Horgan, Catherine Mareiro, Sarah A. Teele

**Affiliations:** From the * Department of Cardiology, Boston Children’s Hospital, Harvard Medical School, Boston, Mass.; † Department of Nursing, Boston Children’s Hospital, Boston, Mass.; ‡ Department of Infection Prevention & Control, Boston Children’s Hospital, Boston, Mass.; § Department of Cardiac Surgery, Boston Children’s Hospital, Boston, Mass.

## Abstract

**Introduction:**

The threshold for ordering blood cultures and initiating empiric broad-spectrum antibiotic therapy is often low in pediatric cardiac intensive care units (CICUs). However, excessive blood cultures carry risks, including anemia, false–positive results, and contamination related to vascular access. Overuse of broad-spectrum antibiotics can lead to the development of multidrug-resistant bacteria.

**Methods:**

In this quality improvement initiative focused on diagnostic stewardship, a blood culture algorithm was developed to reduce unnecessary blood culture sampling in patients with low sepsis probability. All patients admitted to the CICU were included in the study. The preimplementation observation period spanned from January 2022 to June 2023, and the postimplementation observation period lasted from July 2023 to December 2025.

**Results:**

There were 5,958 CICU admissions during the study period, with no statistically significant difference in the baseline clinical characteristics. The number of blood culture samples declined by 26% in the postimplementation period, from 111.9 to 82.8 per 1,000 patient-days (*P* < 0.001), and a centerline shift was observed after the 5-month mark in the statistical process control U-chart. No statistically significant change was observed in the central line–associated bloodstream infection rate per 1,000 line-days (1.7 versus 1.3, *P* = 0.26).

**Conclusions:**

A structured algorithm to support clinicians’ decision-making for blood culture testing resulted in a statistically significant reduction in the number of blood cultures sent. The delayed centerline shift indicated that lasting change required complete practice adoption rather than mere intervention launch. This intervention was not associated with any safety concerns in clinical outcomes.

## INTRODUCTION

Although blood cultures are integral to sepsis management, their overuse in critically ill children represents a form of diagnostic overuse that contributes to patient harm, waste, and device-related complications. Blood culture testing and broad-spectrum antibiotic treatment are often used in children with fever following cardiac surgery.^1–3^ Delayed initiation of broad-spectrum antibiotics is associated with increased morbidity and mortality in patients with active bacterial infections, whereas their overuse promotes the emergence of multidrug-resistant organisms. These considerations highlight the importance of judicious antimicrobial decision-making in pediatric critical care.^3–7^

Diagnostic stewardship has emerged as a set of evidence-based clinical interventions aimed at improving antimicrobial use and reducing antimicrobial resistance by prioritizing the right test for the right patient.^4^ It incorporates elements of antimicrobial stewardship to influence the upstream determinants of clinical decision-making that contribute to unnecessary bacterial testing and antimicrobial treatment. Prior studies on antimicrobial stewardship have demonstrated a sustained reduction in the use of broad-spectrum antimicrobials among pediatric cardiac patients.^5^ In the present study, we sought to adopt an interdisciplinary diagnostic stewardship intervention that would minimize the overuse of blood culture testing in pediatric cardiac intensive care unit (CICU) patients with a low probability of sepsis. We monitored blood culture rates and central line–associated bloodstream infection (CLABSI) rates during an 18-month period before implementation and a 30-month period after implementation.

## METHODS

### Context and interventions

**Fig. 1.**
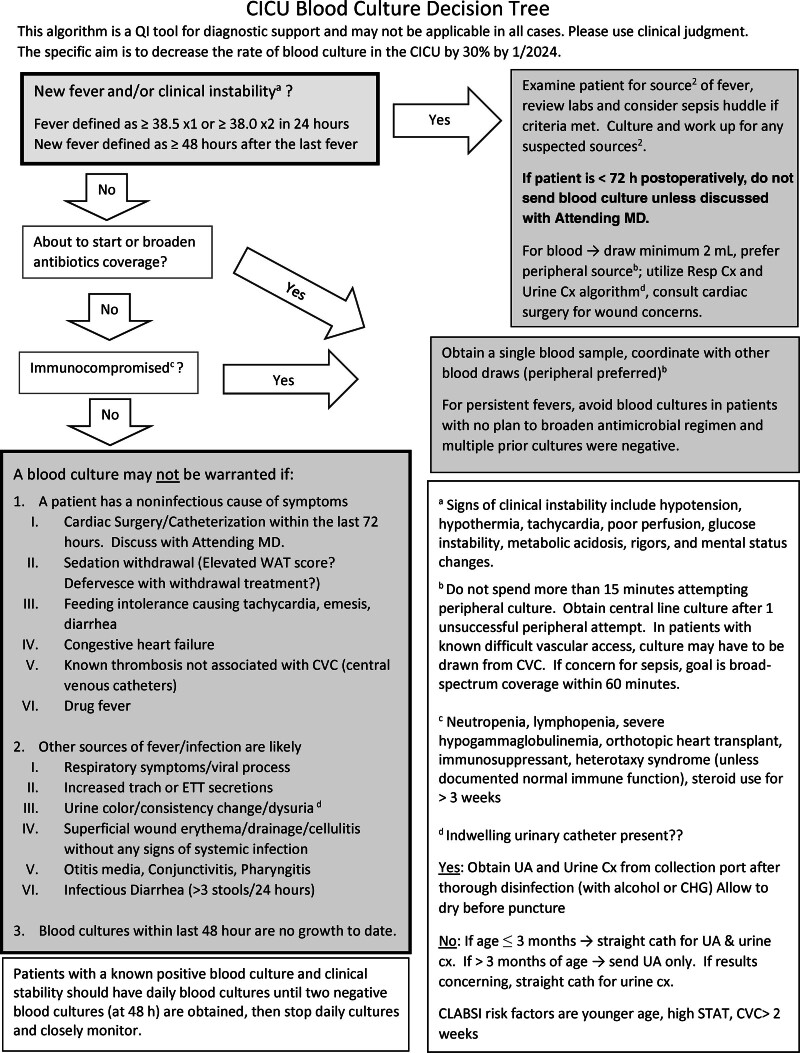
The blood culture decision algorithm. CHG, chlorhexidine gluconate; Cx, culture; UA, urinalysis; WAT, withdrawal assessment tool.

Our CICU formed an infection prevention (IP) team in May 2023. The CICU IP team consisted of a physician, nurses, advanced practice providers, and an IP specialist. Our CICU admission population is heavily weighted toward postoperative cardiac surgical patients with high acuity and complexity necessitating central venous access. As part of a CLABSI reduction initiative, the IP team analyzed blood culture rates and bloodstream infection rates. The team found that providers frequently defaulted to sending blood cultures and initiating broad-spectrum antibiotics early, even in cases of minimal clinical fever. Leveraging a diagnostic stewardship approach, the IP team developed a blood culture decision algorithm intended to safely reduce the frequency of blood culture testing. The algorithm was codesigned with frontline nurses, advanced practice providers, IP specialists, and physicians to ensure it was intuitive, accessible, and integrated into existing workflows. We conducted several educational sessions on the algorithm, gathered feedback, and subsequently revised it. This human-centered approach promoted cross-disciplinary ownership and facilitated sustained adoption. The CICU team and cardiovascular surgeons approved this algorithm in July 2023 (Fig. 1). In this algorithm, we implemented a more stringent fever threshold for blood culture collection. A “new fever” was defined as a single temperature measurement greater than or equal to 38.5 °C or 2 measurements of 38.0 °C within a 24-hour period, occurring at least 48 hours after the resolution of a previous febrile episode. We also focused on 2 populations in the algorithm that were considered low risk for sepsis but were nonetheless subjected to frequent blood culture testing. One group consisted of immediate postcardiac surgery patients (defined as those who had cardiac surgery within the previous 72 h without preexisting increased risk factors), and the other consisted of chronic complex patients with recurrent fever.

Multiple CICU IP initiatives were launched immediately before July 2023. These included (1) weekly safety nosocomial IP rounds identifying patients with indwelling central venous catheters (CVCs) in place for longer than 2 weeks and reviewing their ongoing need with the clinical team; (2) opportunities to convert intravenous medications to enteral administration and reducing the frequency of CVC access (eg, for laboratory draws) were explored; (3) CLABSI prevention bundles, including daily chlorhexidine gluconate treatments for patients older than 2 months, dressing changes, and the use of needleless connectors, were reinforced in accordance with hospital policy; and (4) the explicit assessment of CVC dressings to verify that they are clean, dry, and intact, as well as to discuss the necessity of each CVC with the clinical team.

The preimplementation observation period spanned 18 months, from January 2022 to June 2023, and was immediately followed by a 30-month postimplementation observation period, from July 2023 to December 2025. After July 2022, our CICU bed capacity increased from 31 to 44.

### Measures

Baseline CICU metrics were collected to characterize the patient population, including total admissions, surgical and medical admissions, extracorporeal membrane oxygenation runs, mortality rates, length of stay, patient-days, and indwelling CVC line-days. Observed outcomes included the total number of blood culture specimens sent per patient during the CICU stay, blood cultures obtained within 72 hours after cardiac surgery, and the CLABSI rate.

The primary outcome was the number of blood cultures per 1,000 patient-days. Secondary outcomes included the CLABSI rate per 1,000 line-days. Data are reported according to Standards for Quality Improvement Reporting Excellence (SQUIRE) guidelines.^8^ CLABSI was defined according to the Centers for Disease Control and Prevention guidelines.^9^ The Centers for Disease Control and Prevention added intracardiac lines as eligible central lines for CLABSI in January 2025, and thus were included in our hospital’s central line-days from then on. The blood culture decision algorithm is displayed in Figure 1.

### Analysis

Descriptive data are presented as means with 95% confidence intervals (CI). Chi-square tests were used to compare proportions between outcome groups, and Student’s *T* tests were used to compare continuous variables between groups. All statistical tests were 2-tailed, and a *P* value less than 0.05 was considered significant. Statistical process control (SPC) was performed using U-charts to account for varying monthly subgroup sizes during a 48-month period. We defined the baseline period as the 18 months before algorithm implementation (from January 2022 to June 2023). We compared baseline monthly rates with postimplementation rates during the 30 months following the algorithm’s implementation (from July 2023 to December 2025). A centerline shift was recognized when 8 consecutive data points fell below the baseline mean, indicating a sustained change in the process and the presence of special cause variation. Control limits were calculated using standard SPC U-chart formulas. All analysis and process control chart construction were performed with Excel (version 16.77.1, Microsoft, Redmond, WA).

### Ethical Considerations

The institutional review board of Boston Children’s Hospital reviewed this quality improvement project and determined that this project did not meet the criteria for human subject research (IRB-P00053261).

## RESULTS

### CICU Characteristics

There were 5,958 CICU admissions during the entire period (January 2022–December 2025). In Table 1, we compare the clinical characteristics between the preimplementation period (January 2022–June 2023) and the postimplementation period (July 2023–December 2025). There was no statistical difference in mean monthly rate of total CICU admissions (122 versus 125, *P* = 0.41), surgical admissions (78 versus 78, *P* = 0.92), medical admissions (44 versus 48, *P* = 0.19), extracorporeal membrane oxygenation use (4.2 versus 3.7, *P* = 0.45), mortality (2.9 versus 2.8, *P* = 0.7), CICU patient-days (1040 versus 1080, *P* = 0.24), and CVC days (763 versus 783, *P* = 0.58), and in median CICU length of stay (3.9 versus 3.9, *P* = 0.97) between the 2 periods.

**Table 1.** Baseline Clinical Characteristics of CICU

Outcome	Mean Monthly Rate (95% CI)		*P*
	Preimplementation (January 2022–June 2023)	Postimplementation (July 2023–December 2025)	
Total CICU admissions per month	122 (115–129)	125 (121–130)	0.41
Surgical admissions per month	78 (72–84)	78 (74–82)	0.92
Medical admissions per month	44 (40–48)	48 (44–51)	0.19
ECMO per month	4.2 (3.3–5.1)	3.7 (3.1–4.4)	0.45
Mortality per month	2.9 (2.3–3.5)	2.8 (2.4–3.2)	0.7
Median CICU length of stay	3.9 (3.6–4.2)	3.9 (3.7–4.1)	0.97
CICU patient-days per month	1040 (978–1102)	1080 (1045–1115)	0.24
CVC days per month	763 (721–806)	783 (735–832)	0.58

ECMO, extracorporeal membrane oxygenation.

### Statistical Process Control

**Fig. 2.**
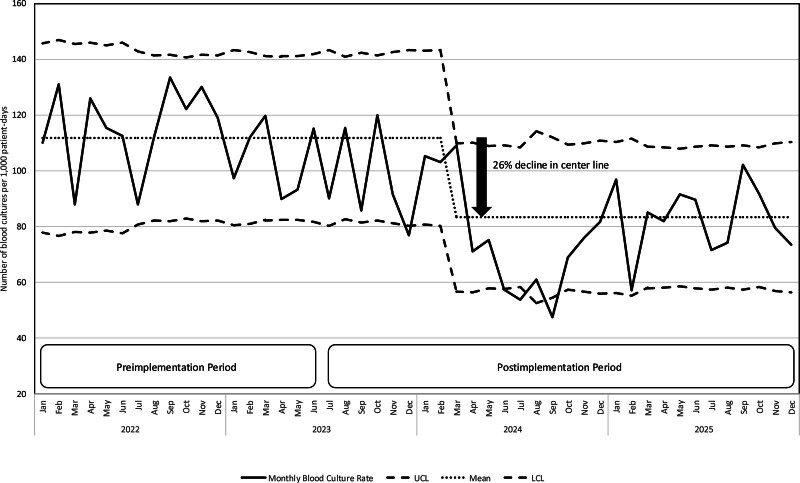
U-chart for monthly blood culture per 1,000 patient-days across 48 months. LCL, lower control limit; UCL, upper control limit.

**Fig. 3.**
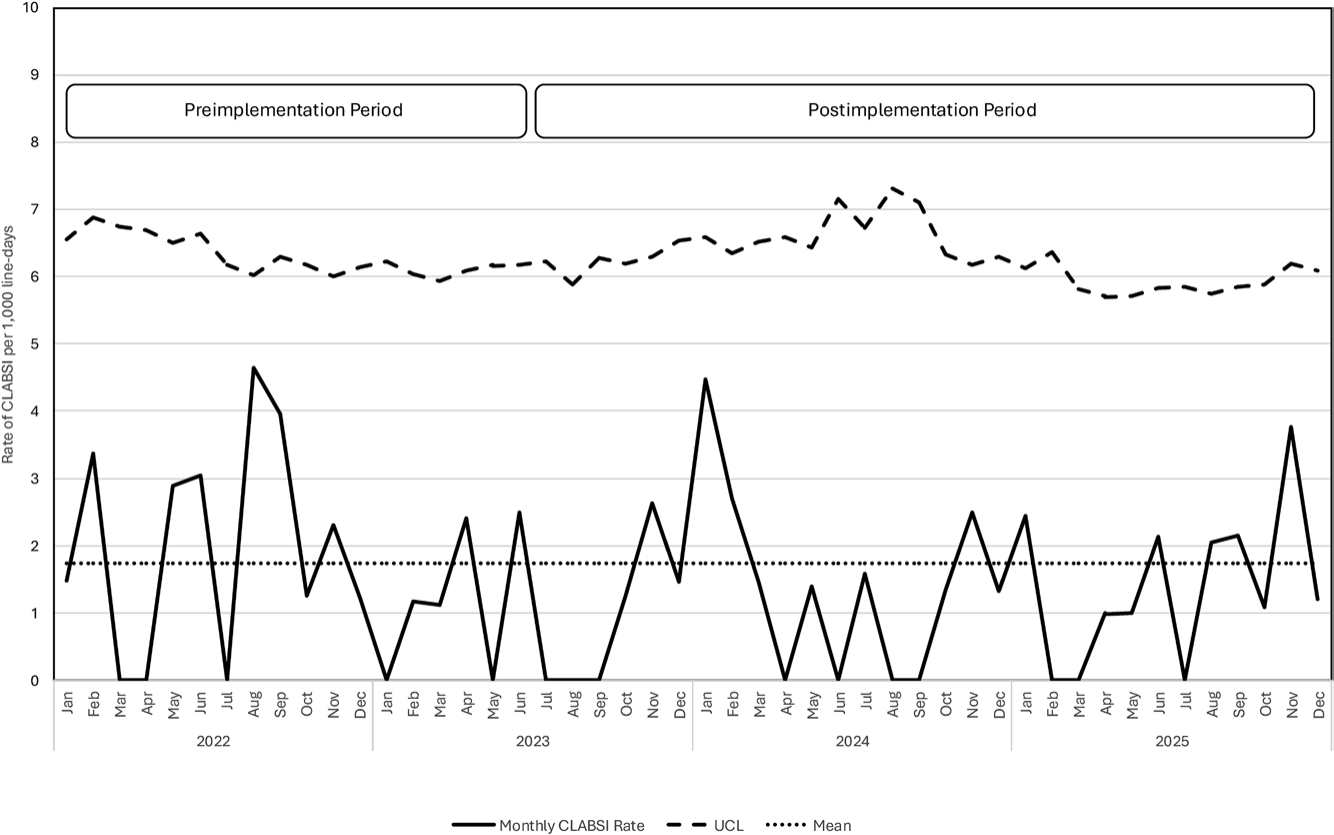
U-chart for monthly CLABSI per 1,000 line-days across 48 months.

Our primary outcome, the rate of monthly blood cultures per 1,000 patient-days, is displayed in the SPC U-chart (Fig. 2). The center line shift was observed after the 5-month mark, declined by 26%, and was sustained throughout the postimplementation period. During the “Study” phase of the Plan-Do-Study-Act cycle, the lack of an immediate decline in culture rates indicated that the unit’s large scale and staff headcount led to an initially incomplete dissemination of the intervention. This observation proved more instructive than aggregated statistical outcomes, as it highlighted the human-factor challenges inherent in large-scale clinical change. By responding in the “Act” phase with targeted bedside training and case-based methodology, the team successfully facilitated a robust adoption of the protocol. The measurable improvements after the 5-month mark validate the importance of iterative evaluation and the need to address implementation fidelity to achieve meaningful clinical shifts. The secondary outcome, CLABSI rate per 1,000 line-days, is displayed in the SPC U-chart (Fig. 3). No center line shift was observed throughout the postimplementation period.

### Primary and Secondary Outcomes

Table 2 summarizes the comparison of outcomes. The rate of monthly blood culture tests per 1,000 patient-days in the preimplementation period was significantly higher than in the postimplementation period (111.9 versus 82.8, *P* < 0.001). It declined by 26% from 111.9 to 82.8 cultures per 1,000 patient-days in the postimplementation period (relative rate, 0.74; 95% CI, 0.73–0.75). The mean monthly rate of blood culture tests within 72 hours after cardiac surgery declined by 59% from 8.7 to 3.6 cultures per month in the postimplementation period (relative rate, 0.41; 95% CI, 0.38–0.43). In contrast, no statistically significant differences were noted in the CLABSI rate per 1,000 line-days and blood culture positivity rate. We did not define a chronic complex patient; therefore, we did not perform any analyses on the subgroup.

**Table 2.** Primary and Secondary Clinical Outcomes

Outcome	Mean Monthly Rate (95% CI)		Postimplementation Versus Preimplementation		*P*
	Preimplementation (January 2022–June 2023)	Postimplementation (July 2023–December 2025)	Relative Rate (95% CI)	Absolute Rate Difference (95% CI)	
Blood cultures per 1,000 patient-days	111.9 (105.0–118.8)	82.8 (76.2–89.3)	0.74 (0.73–0.75)	−29.1 (−28.7 to −29.5)	<0.001
CLABSI per 1,000 line-days	1.7 (1.1–2.4)	1.3 (0.9–1.7)	0.75 (0.71–0.82)	−0.44 (−0.19 to −0.69)	0.26
Positive blood culture rate, including contamination, %	9.7 (6.9–12.4)	8.6 (6.1–11.1)	0.89 (0.88–0.89)	−1.1 (−0.84 to −1.32)	0.59
The number of blood cultures obtained within 72 h postcardiac surgery	8.7 (7.2–10.3)	3.6 (2.8–4.4)	0.41 (0.38–0.43)	−5.1 (−4.4 to −5.8)	<0.001

## DISCUSSION

This single-center process improvement initiative, which leveraged diagnostic stewardship, was associated with a reduction in blood culture sampling among pediatric CICU patients with a low probability of sepsis. Following the introduction of a blood culture decision algorithm, the monthly blood culture testing rate at our center decreased by 26% relative to baseline, with a 5-month delay in centerline shift. This delayed centerline shift highlighted a fundamental truth of clinical implementation: practice adoption is not instantaneous. Prioritizing the identification of implementation gaps over immediate statistical results is essential for driving meaningful and long-term practice change. The CLABSI rate remained unchanged during the study period (Fig. 3). This intervention was not associated with any concerning changes in safety outcomes, such as mortality or length of stay in the CICU. Our findings, therefore, demonstrate the efficacy of an interdisciplinary diagnostic stewardship approach in optimizing blood culture sampling and empiric antibiotic use.

Blood culture tests are among the most ordered microbiological tests in hospitalized patients, and defining their appropriateness remains a major hurdle in clinical practice. Diagnostic stewardship principles have introduced fever algorithms to reduce unnecessary blood cultures in critically ill children.^10,11^ In the first multicenter collaborative of its kind, the Bright STAR (Testing Stewardship for Antibiotic Reduction) Collaborative developed a diagnostic stewardship collaboration to optimize blood culture practices in critically ill children without suspected sepsis. Using a variety of clinical decision support tools, the program ultimately decreased blood culture rates by 33% and CLABSI rates by 36% across 14 participating pediatric intensive care units in the United States.^11^ Our findings expanded on those from the Bright STAR initiative by offering a more targeted approach in pediatric CICU patients, the majority of whom were postoperative. In our study, we highlighted 2 specific populations with a low probability of sepsis, who thus represented low-value applications of bacterial blood tests. One group consisted of immediate postcardiac surgery patients (defined as those who had cardiac surgery within the past 72 h), and the other consisted of chronic complex patients with recurrent fever. Our study showed that the mean monthly rate of blood culture tests within 72 hours postcardiac surgery declined by 59%. By focusing our study intervention on patients considered the lowest risk, we significantly improved (reduced) blood culture rates without a concomitant increase in the incidence of adverse outcomes. We were unable to identify a specific dataset for chronic complex patients because no formal definition was established for this study. We have noted this as an area for future study.

Although our center’s CLABSI rate improved simultaneously, it did not reach the average rate reported in prior studies. The aggregated CLABSI rate in multiple pediatric CICUs was recently reported as 1.1 per 1,000 line-days.^12^ Significant risk factors for CLABSI in children in the CICU were younger age, greater surgical complexity, and total catheter days.^13,14^ Our CLABSI rate in the preimplementation period was 1.7 per 1,000 line-days, which declined to 1.3 per 1,000 line-days in the postimplementation period, slightly above the noted benchmark target. However, no centerline shift was observed in the SPC U-chart (Fig. 3). We used tools and reinforced behaviors such as CVC maintenance bundles, daily discussion of the CVC need, limiting CVC entries, disinfecting needle connectors, changing the CVC dressing every 7 days, and replacing connectors and administration sets according to hospital policy, before the postimplementation period.^15^

Diagnostic stewardship for blood culture use is essential to help mitigate multiple potential harms beyond CLABSI prevention. For example, blood conservation can also be improved with effective blood culture stewardship. A single blood culture bottle typically requires 1–3 mL of blood volume for optimal diagnosis.^16^ Unnecessary blood draws can contribute to iatrogenic anemia, particularly in neonates, in whom even minimal blood loss may be clinically significant. Additionally, excessive blood cultures are associated with increased risks of false-positive results and vascular access contamination, potentially leading to unwarranted interventions and adverse outcomes. Our algorithm was developed with strong consideration for human factors to ensure it was intuitive, easily accessible, and seamlessly integrated into clinical workflows. Its simple design allows clinicians to quickly interpret and apply recommendations without disrupting existing care processes. By aligning it with routine clinical decision-making, it promotes consistent, evidence-based practice at the point of care. Furthermore, integrating diagnostic and antimicrobial stewardship principles into the algorithm supports the appropriate use of tests and antimicrobials, reinforcing a coordinated approach to IP. This human-centered, workflow-compatible design enhances adoption, sustainability, and overall effectiveness in reducing healthcare-associated infections such as CLABSI.

## LIMITATIONS

Although our study demonstrates a significant reduction in blood culture sampling, establishing performance benchmarks is difficult. We cannot definitively determine the proportion of our reduction that specifically targeted low-value testing. Our study was a single-center quality improvement initiative limited to a CICU cohort and used internal resources. Our approach to this work might not be generalizable to other CICUs with different programmatic philosophies and workflows. The algorithm was not established as a strict protocol, and the bedside clinician’s judgment ultimately dictated the clinical decisions; many of the measured outcomes have some degree of interpractitioner variability. These points underscore the need for multi-institutional efforts to define clinically necessary testing.

Specific balancing measures and datasets were not predefined for this study. Although septic shock is a potential complication of late diagnosis, these outcomes were not collected. Several initiatives were underway simultaneously at both the unit and hospital levels to reduce CLABSIs, which might have confounded the outcomes. During the study period, the institution transitioned to a new electronic medical record system; as a result, standardized antimicrobial stewardship metrics, including days of therapy, were not consistently captured or available for extraction.

## CONCLUSIONS

In our single-center, high-volume pediatric CICU, an interdisciplinary diagnostic stewardship program successfully reduced the monthly blood culture testing rate by 26% relative to baseline, with a 5-month delay in centerline shift. The delayed centerline shift indicated that lasting clinical change required complete practice adoption rather than mere intervention launch. The CLABSI rate remained unchanged. This intervention was not associated with safety concerns in clinical outcomes.
